# Cerebral air embolism during commercial air travel due to a giant pulmonary bulla

**DOI:** 10.1093/jscr/rjag955

**Published:** 2026-10-22

**Authors:** Jeevan Francis, Saima Azam, Mohammed Wesam Khalil, Keith Buchan

**Affiliations:** Department of Cardiothoracic Surgery, Aberdeen Royal Infirmary, Foresterhill Road, Aberdeen AB25 2ZN, United Kingdom; Faculty of Medicine, King’s College London, London, United Kingdom; Department of Cardiothoracic Surgery, Aberdeen Royal Infirmary, Foresterhill Road, Aberdeen AB25 2ZN, United Kingdom; Department of Cardiothoracic Surgery, Aberdeen Royal Infirmary, Foresterhill Road, Aberdeen AB25 2ZN, United Kingdom; Department of Cardiothoracic Surgery, Aberdeen Royal Infirmary, Foresterhill Road, Aberdeen AB25 2ZN, United Kingdom

**Keywords:** air embolism, air travel, pulmonary bullae, thoracic surgery

## Abstract

Cerebral air embolism is a rare but potentially catastrophic complication of air travel in patients with pulmonary bullae or other air-containing intrathoracic lesions. A 59-year-old man lost consciousness approximately 20 min after take-off on a commercial flight. The aircraft immediately descended and returned to its departure airport, where he was transferred for emergency assessment. Computed tomography of the head demonstrated air bubbles in several intracranial cerebral arteries consistent with cerebral arterial air embolism, while thoracic imaging identified a giant right upper-lobe bulla. He recovered with mild persistent right-sided hemiparesis and subsequently underwent resection of the bulla. This case demonstrates how the reduction in cabin pressure during an otherwise routine flight may cause expansion of a pulmonary bulla, pulmonary barotrauma and entry of air into the systemic circulation. Cerebral air embolism should be considered when abrupt neurological symptoms occur during or shortly after aircraft ascent.

## Introduction

Commercial aircraft cruise at altitudes where the external atmospheric pressure is incompatible with normal human physiology. Aircraft cabins are therefore pressurized, although usually only to an equivalent altitude of 6000–8000 feet. At a cabin altitude of 8000 feet, atmospheric pressure is ~565 mm Hg, compared with approximately 760 mm Hg at sea level. This reduction is generally well tolerated but causes enclosed gas to expand according to Boyle’s law: 


$$ P_{1}V_{1} = \mathrm{P}_{2}V_{2} $$

Accordingly, a fixed volume of gas at sea level may expand by ~30%–35% during ascent to normal cruising cabin pressure. Most passengers experience no adverse consequences because gas within the lungs communicates freely with the atmosphere. However, gas contained within a non-communicating pulmonary bulla or cyst may be unable to escape. Expansion of such a lesion can damage its wall or the adjacent lung and, rarely, allow air to enter the pulmonary circulation [1–3]. Written informed consent was obtained from the patient for publication of this case report and the accompanying images.

## Case presentation

A 59-year-old man was travelling on a commercial aircraft when he suddenly lost consciousness approximately 20 min after departure. The timing of the event coincided with the period of aircraft ascent and reduction in cabin pressure. The aircraft descended and eventually returned to its departure airport to allow urgent medical assessment.

Emergency computed tomography (CT) imaging of the head demonstrated intracranial air within the cerebral circulation, consistent with cerebral arterial air embolism. Chest imaging performed during the same assessment showed a giant bulla occupying the right upper lobe (Fig. 1). The combination of an acute neurological event during aircraft ascent, visible intracranial air and a large air-containing pulmonary lesion supported a diagnosis of cerebral air embolism caused by pulmonary barotrauma.

**Figure 1:**
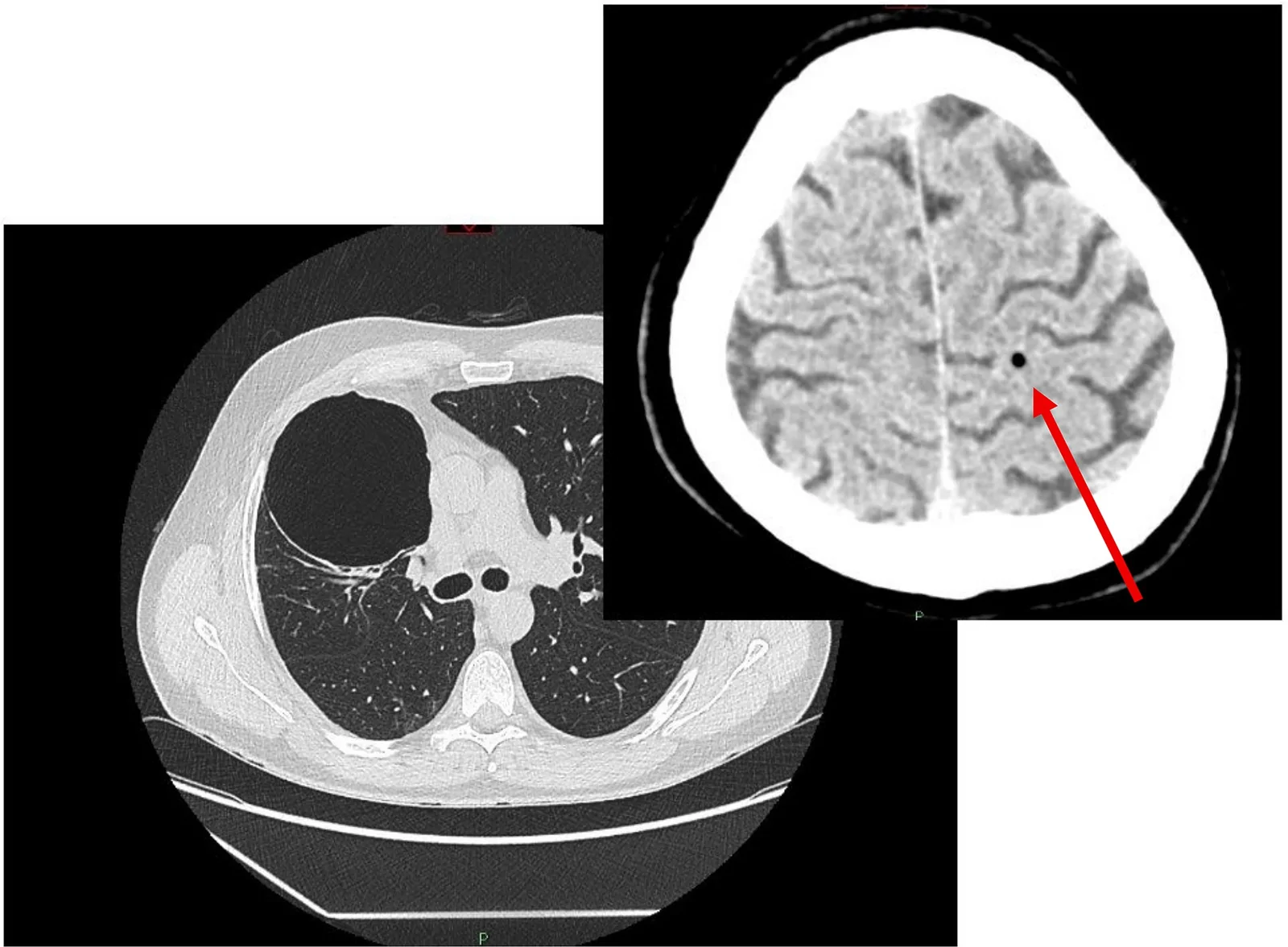
Composite CT of head and chest showing cerebral air embolism (arrow) and a giant pulmonary bulla which was subsequently resected.

The patient survived the event and made a good neurological recovery, although mild right-sided hemiparesis persisted. The giant pulmonary bulla was subsequently resected to remove the source of the air embolism and prevent future complications.

## Discussion

Cerebral air embolism during commercial air travel is extremely rare. Reported cases have usually involved an underlying pulmonary bulla, bronchogenic cyst, congenital pulmonary lesion or postoperative air-containing cavity [4–6]. Symptoms characteristically develop during or shortly after ascent, when falling cabin pressure produces the greatest expansion of trapped gas. Presentations range from headache, visual disturbance and focal weakness to seizures, loss of consciousness, major stroke, and death.

For cerebral arterial embolization to occur, air must gain access to the systemic circulation. Expansion of a bulla may stretch or rupture its wall and injure neighbouring lung tissue. If a communication develops between the air space and adjacent pulmonary venules, the pressure gradient may force air into the pulmonary venous circulation. Air can then pass through the left heart and enter the systemic arteries without an intracardiac shunt. Cerebral vessels are particularly vulnerable. Gas bubbles cause immediate mechanical obstruction but may also produce endothelial injury, platelet activation, inflammation and secondary cerebral oedema, extending injury beyond the period of initial vascular occlusion [7]. This is distinct from decompression sickness, commonly known as ‘the bends’, in which nitrogen dissolved within the tissues under pressure comes out of solution during ascent. The British Thoracic Society considers pulmonary bullae and cysts contraindications to diving because of the risk of pulmonary rupture, pneumothorax, and systemic gas embolism.

Guidance for commercial air travel is less definitive. The 2022 British Thoracic Society Clinical Statement recognizes that the cabin pressure at an equivalent altitude of 8000 feet produces ~38% expansion of humidified trapped gas. It recommends specialist assessment for passengers with cystic lung disease, chronic intrathoracic air spaces or an increased risk of pneumothorax and identifies bronchogenic cysts as a contraindication to flying. Pulmonary bullae are not addressed specifically. Given the potentially fatal cases reported, a giant pulmonary bulla should nevertheless prompt specialist assessment, including hypobaric testing at a regional hyperbaric medicine facility. Air travel should be avoided when there has been previous barotrauma or cerebral air embolism until definitive treatment has been undertaken.

The diagnosis requires a high index of suspicion. Sudden focal neurological impairment or loss of consciousness during a flight may initially be attributed to thromboembolic stroke, seizure, syncope, hypoglycaemia or hypoxaemia. The close relationship to aircraft ascent should raise the possibility of barotrauma, particularly when there is known cystic or bullous lung disease. Intracranial air on CT is strongly supportive, although cerebral gas may be absorbed rapidly and its absence on delayed imaging does not exclude the diagnosis. Thoracic imaging is essential to identify a pulmonary source and to assess for associated pneumothorax, pulmonary haemorrhage or other barotraumatic injury.

Immediate management of suspected arterial gas embolism includes resuscitation and administration of 100% oxygen. A high inspired oxygen concentration treats hypoxaemia and increases the gradient for nitrogen to leave the bubble, thereby accelerating its reduction. Hyperbaric oxygen therapy is recommended for gas embolism with neurological or cardiovascular manifestations. It reduces bubble volume through recompression while increasing dissolved oxygen delivery to ischaemic tissue. Treatment should be arranged as early as possible, although neurological improvement has been reported even when hyperbaric treatment is delayed [8]. In our patient’s case, the pilot’s decision to return immediately to the departure airport may have been life-saving as it would have limited the patient’s exposure to cerebral air embolism.

The pulmonary lesion must be addressed once the patient is stable. Definitive resection prevents recurrence by removing the risk of further bulla expansion, rupture, pneumothorax or embolization. Until the lesion has been assessed and appropriately treated, further exposure to substantial changes in ambient pressure should be avoided.

Current guidance does not specifically classify pulmonary bullae as an absolute contraindication to commercial air travel. However, patients with a giant bulla, previous pressure-related symptoms or other evidence of an enclosed pulmonary air space should undergo specialist assessment and should generally avoid flying until the lesion has been definitively treated.
